# Embryonic Exposure to Cigarette Smoke Extract Impedes Skeletal Development and Evokes Craniofacial Defects in Zebrafish

**DOI:** 10.3390/ijms23179904

**Published:** 2022-08-31

**Authors:** Omran Karmach, Joseph V. Madrid, Subham Dasgupta, David C. Volz, Nicole I. zur Nieden

**Affiliations:** 1 Department of Molecular, Cell & Systems Biology, College of Natural and Agricultural Sciences, University of California Riverside, Riverside, CA 92521, USA; 2 Stem Cell Center, College of Natural and Agricultural Sciences, University of California Riverside, Riverside, CA 92521, USA; 3 Department of Environmental Sciences, College of Natural and Agricultural Sciences, University of California Riverside, Riverside, CA 92521, USA

**Keywords:** developmental toxicity, neural crest, osteoblasts, cigarettes, tobacco smoke solution, craniofacial skeleton

## Abstract

Exposure to cigarette smoke represents the largest source of preventable death and disease in the United States. This may be in part due to the nature of the delayed harmful effects as well as the lack of awareness of the scope of harm presented by these products. The presence of “light” versions further clouds the harmful effects of tobacco products. While active smoking in expectant mothers may be reduced by educational and outreach campaigns, exposure to secondhand smoke is often involuntary yet may harm the developing embryo. In this study, we show that the main component of secondhand smoke, sidestream cigarette smoke, from several brands, including harm-reduction versions, triggered unsuccessful hatching at 3 dpf and reduced overall survival at 6 dpf in developing zebrafish. At non-lethal concentrations, craniofacial defects with different severity based on the cigarette smoke extract were noted by 6 dpf. All tested products, including harm-reduction products, significantly impacted cartilage formation and/or bone mineralization in zebrafish embryos, independent of whether the bones/cartilage formed from the mesoderm or neural crest. Together, these results in a model system often used to detect embryonic malformations imply that exposure of a woman to secondhand smoke while pregnant may lead to mineralization issues in the skeleton of her newborn, ultimately adding a direct in utero association to the increased fracture risk observed in children of mothers exposed to cigarette smoke.

## 1. Introduction

Tobacco products are amongst the most studied harmful chemical mixtures with a detrimental impact on human health [1,2,3]. Among the many tissues targeted by toxicants in tobacco products are a number of skeletal sites, including the axial, appendicular, and cranial skeleton [4,5,6,7,8,9]. In smokers, all of these sites exhibit significantly reduced bone mass compared to non-smokers, and specifically, the risk to incur a vertebral fracture is increased [10,11,12]. Furthermore, smokers lost significantly more bone mass over time compared to non-smokers [12,13]. This outcome is exaggerated in postmenopausal women smokers, who have diminished bone density compared to postmenopausal non-smoking women [14]. Resultant hip fractures positively associate with an increasing age of the smoker [10,12]. Similarly, osteopenia and osteoporosis rates were significantly higher in smokers, with lower levels of antioxidant enzymes and elevated oxidative stress also observed [12,14].

While exposure to firsthand smoke, which is the smoke the consumer inhales, is an individual risk that may be lessened with proper education and outreach, environmental or secondhand smoke is of additional concern as exposure is often involuntary. Approximately 85% of the secondhand smoke is sidestream (SS) smoke, either in its unprocessed form when it is expelled from the tip of the cigarette, or the exhaled form processed through the lungs of the smoker [15,16]. Since 1964, about 2.5 million non-smokers have died from health ailments provoked by exposure to secondhand smoke [17]. While smokefree laws have reduced exposure in public places, for many, especially children and pregnant women, exposure to secondhand smoke occurs in private locations such as homes and vehicles [18] where regulations are difficult to implement. 

To test for the potency of secondhand smoke to interfere with the development of bones, we recently employed an in vitro osteogenesis model based on human embryonic stem cells (hESCs). In a prior study [19], we tested SS smoke as well as mainstream (MS) smoke, which is directly inhaled by the smoker from the butt of the cigarette and makes up firsthand smoke. Due to SS smoke being a major component of secondhand smoke, it acted as a surrogate for environmental cigarette smoke exposure. Among all five tested products, SS smoke was always more developmentally toxic to differentiating osteoblasts than the MS smoke from the same brand of cigarette [19], underscoring the need to illuminate skeletal toxicity mediated by SS smoke exposure in more detail, which we set out to do in the current study.

In addition, our previous study contrasted the toxicity of conventional cigarette smoke extracts to those from cigarettes previously marketed as “light” versions of the parent brand. This was because many women may turn to these “harm reducing” tobacco products as a “safe” alternative, especially during pregnancy. The “harm reduction” denominator stems from the comparatively low nicotine and nitrosamine content, yet both in vitro and in vivo research showed that harm-reduction products still cause significant harm during embryonic development [20]. Particularly in our skeletal hESC model, SS smoke extracts from Camel Blue and Marlboro Gold elicited defects to osteoblast differentiation at concentrations that allowed continued cellular survival [19]. 

While ESCs are ideal for studying toxicant effects on specific differentiation pathways in vitro and how these toxicants might alter gene expression and protein levels to change cell fate decisions, ESC-derived cell types lack interactions with other tissues or organ systems present in a whole organism. Zebrafish, in turn, offer tissue and organ interactions missing from ESCs, in addition to the large offspring yield, embryo transparency, and metabolism at later stages of development. Zebrafish, like humans, develop bones through intramembranous ossification, where mesenchymal cells condense and differentiate into osteoblasts, as well as endochondral ossification, where mesenchymal cells condense and differentiate into chondrocytes to form a cartilage template that is replaced by bone [21]. Due to the presented limitations of the human in vitro model and advantages of zebrafish, the objective of this study was to confirm the adverse skeletal outcome associated with sidestream smoke encounters in a whole-animal exposure regimen using the teleost. 

## 2. Results

### 2.1. Sidestream Tobacco Smoke Solutions Alter Zebrafish Embryo Survival and Hatching In Vivo

**Figure 1:**
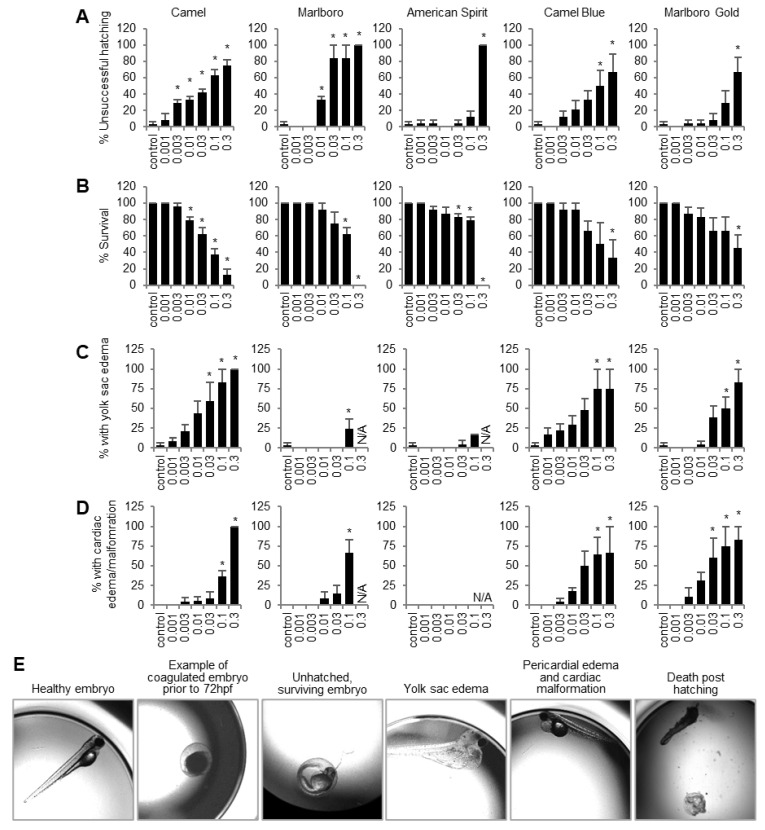
Tobacco products cause early developmental problems in zebrafish embryos. Embryos were placed in E3 embryo media containing tobacco smoke solution at varying concentrations at 5 hpf and assessed at 3 dpf and 6 dpf. Hatching success (3 dpf) (**A**) and survival (6 dpf) (**B**) was measured out of the original 24 embryos. Yolk sac edema (**C**) or defects in heart development (**D**) were assessed in the surviving larvae. X-axes represent concentrations in puff equivalents (PE). (**E**) Example images of phenotypes scored in (**A**–**D**). * *p* < 0.05, one-way ANOVA with a Dunnett’s post hoc test to correct for multiple comparisons. N/A, not assessed.

A zebrafish study was designed to evaluate the potential harmful effects of developmental exposure to tobacco in an animal model that exhibits metabolic activity, a feature that in vitro cell systems are lacking. Both survival endpoints as well as osteogenesis endpoints previously assessed in our human in vitro study to distinguish general cytotoxicity from true developmental defects [19] were also included here. By 72 hpf, embryos were assessed for successful dechorionation, but not all embryos had hatched properly (Figure 1A). Camel treatment showed a significant increase in unsuccessful hatching starting at 0.003 puff equivalents (PE), an effect that worsened with increased Camel concentrations. Marlboro treatment significantly increased hatching failure one concentration up from Camel, at 0.01 PE. Camel Blue and Marlboro Gold showed a trend positively correlating with concentration but that was only significant at 0.1 and 0.3 PE, respectively. American Spirit significantly increased unsuccessful hatching at 0.3 PE.

As for mortality, all tobacco products, including harm-reduction products, significantly reduced zebrafish larvae survival at 6 dpf. Specifically, Camel, between concentrations of 0.01 PE and 0.3 PE, significantly reduced the survival rate (Figure 1B). Marlboro significantly reduced the survival rate at 0.1 PE and 0.3 PE and American Spirit significantly reduced the survival rate at 0.03 PE, 0.1 PE, and 0.3 PE. At the highest concentration for both American Spirit and Marlboro, none of the larvae survived. The “harm-reducing” Camel Blue and Marlboro Gold showed a significant reduction in survival rate at the highest concentration tested (0.3 PE) only.

### 2.2. All Tobacco Products, Including Harm-Reduction Products, Significantly Increased General Developmental Defects In Vivo

In addition to impacting embryo hatching and survival, larvae from all treatments showed signs of increased yolk sac edema at 6 dpf (Figure 1C); Camel exposure significantly increased the percentage of larvae with yolk sac edema between 0.03 PE and 0.3 PE. Marlboro and American Spirit only showed significant increases in yolk sac edema at 0.1 PE. Edema could not be assessed at 0.3 PE for these two treatments, because none of the embryos had survived. Camel Blue began trending at 0.03 PE and was significant at 0.1 PE and 0.3 PE, as was also seen for Marlboro Gold.

Additionally, most treatments caused an enlargement in the embryo heart. Camel and Camel Blue showed significant increases in the number of larvae with an enlarged heart at 0.1 PE and 0.3 PE (Figure 1D). Marlboro only showed a significant increase at 0.1 PE, while Marlboro Gold trended positively with a significant increase from 0.03 PE to 0.3 PE. American Spirit was the only treatment that did not show signs of heart enlargement.

### 2.3. Both Conventional and Harm-Reduction Tobacco Products Affect Cartilage Formation

In both humans and zebrafish, bone is formed by either intramembranous bone formation or endochondral bone formation [22,23]. Endochondral bone formation is the more prevalent of the two and it begins with the formation of a cartilage template that is replaced by osteoprogenitor cells that differentiate into osteocytes within a calcified matrix [24]. Thus, to better understand the effect of the tobacco products on the skeleton, cartilage formation was first examined.

**Figure 2:**
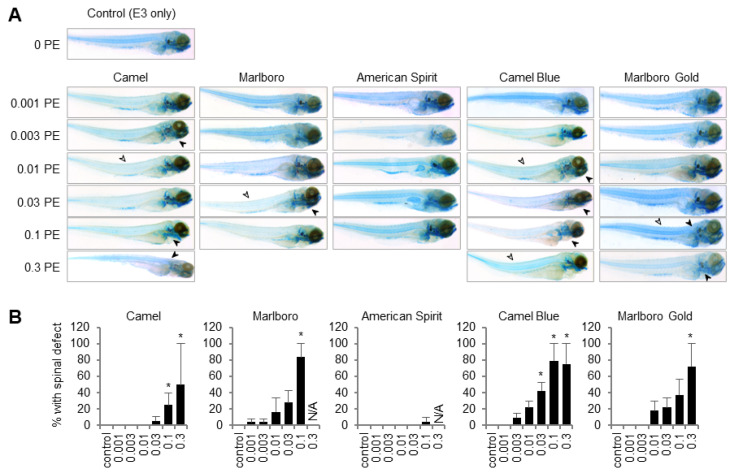
Tobacco products affected cartilage development in zebrafish. Embryos were placed in a tobacco smoke solution containing E3 embryo media at 5 hpf and collected, fixed, and stained with Alcian Blue at 6 dpf. (**A**) Representative images of Alcian Blue staining assessed with a light microscope. Note that larvae were oriented according to the location of the sac with respect to the body axis. Spine curvature is denoted with open arrowheads, twisted spines (in relation to the head) are denoted with closed arrowheads. (**B**) Defects in spinal development were assessed in the surviving embryos, *n* = 24. X-axis gives SS tobacco smoke concentrations in puff equivalents (PE). * *p* < 0.05, one-way ANOVA with a Dunnett’s post hoc test to correct for multiple comparisons. N/A, not assessed; PE, puff equivalent.

Upon morphological observation of the embryos post treatment, it was noted that some treatments affected the curvature of the spine of the embryos more than others (open arrowheads, Figure 2A). In addition, some embryos exhibited severe twisting of the head in relation to the body axis (closed arrowheads, Figure 2A). These malformations were summarized as spinal defects and quantified for the respective exposures and concentrations (Figure 2B). Such quantifications revealed that spinal defects were numerous in SS smoke of all tobacco products, except for American Spirit, and increased in a concentration-dependent manner. No differences were noted between conventional and harm-reduction products.

**Figure 3:**
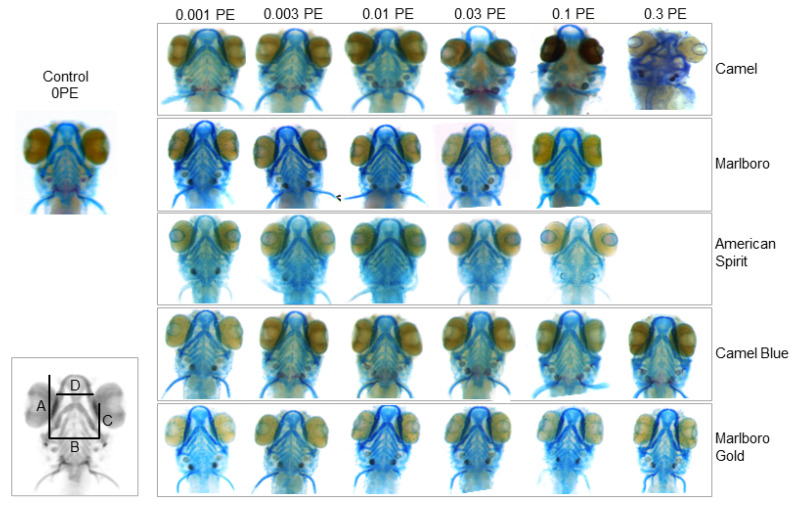
Effect of tobacco products on craniofacial cartilage development in exposed embryos. Embryos were placed in a tobacco smoke solution (in E3 embryo media) at 5 hpf and collected, fixed, and stained with Alcian Blue at 6 dpf. Representative images of the surviving larvae heads, *n* = 24. (Inset) Schematic of the parameters measured to quantitatively assess cartilage defects. Distance A: from the left hyosymplectic to the top of anterior limit. Distance B: from the left hyosymplectic to the right hyosymplectic. Distance C: from the right hyosymplectic to the top of ceratohyal. Distance D: from the right articulation to the left articulation (compare Figure 4).

Similarly, craniofacial cartilage architecture appeared more severely malformed in some tobacco exposures than others (Figure 3). Specifically, at the highest Camel exposure, major malformations of the cranial cartilage scaffold were noted.

Indeed, other investigations have linked tobacco smoke components to the compromise of bone development [25,26]. Thus, the decrease in calcification seen in human osteogenic ESC cultures treated with tobacco products [19] as well as the observed spinal curvature and twisting prompted us to further investigate bone and cartilage formation in the exposed zebrafish larvae.

**Figure 4:**
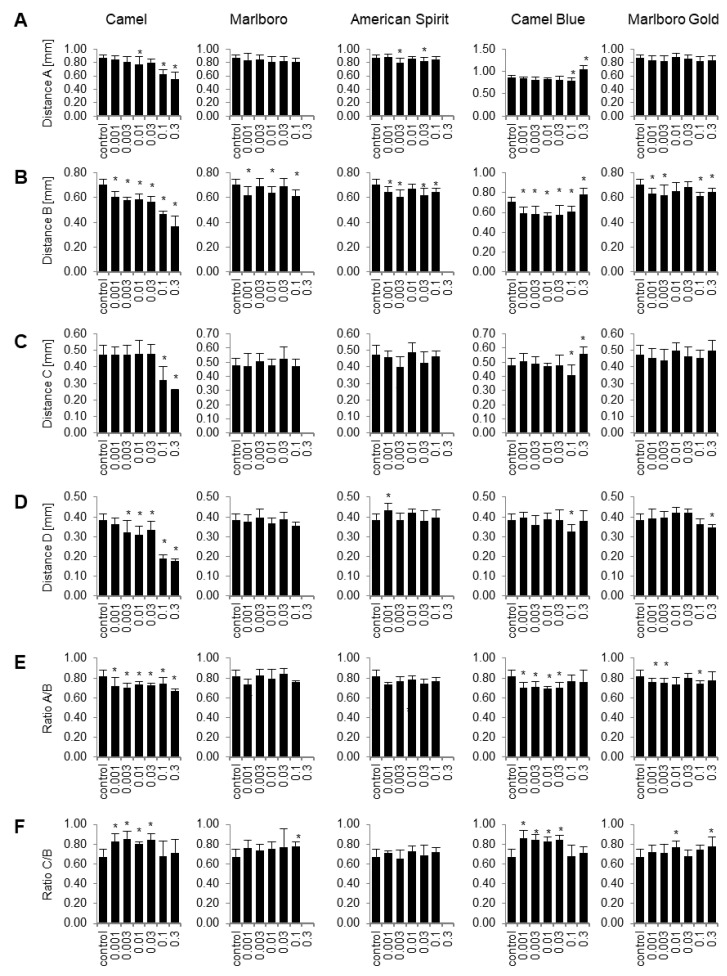
Quantification of the effect of tobacco smoke extracts on cartilage development in zebrafish. Embryos were placed in E3 embryo media containing tobacco smoke solutions at the concentrations indicated at 5 hpf and stained with Alcian Blue at 6 dpf. The length of 4 parameters shown in the inset in Figure 3 were quantitatively measured from images. (**A**) Distance A: from the left hyosymplectic (h) to the top of anterior limit (an). (**B**) Distance B: from the left hyosymplectic (h) to the right hyosymplectic (h). (**C**) Distance C: from the right hyosymplectic (h) to the top of ceratohyal (ch). (**D**) Distance D: from the right articulation (ar) to the left articulation (ar). Both A and C measurements were then compared to distance B with A/B (**E**) and C/B ratios (**F**), respectively. X-axis units are given in puff equivalents (PE). * *p* < 0.05, one-way ANOVA with a Dunnett’s post hoc test to correct for multiple comparisons.

To assess the damage done to the cartilage architecture also in specimen in which architectural malformations were not obvious upon morphological inspection, we employed image morphometry to assess cartilage parameters quantitatively (Figure 4, compare schematic in Figure 3). Together, these appraised the width and length of the skulls as well as their relations. Camel significantly reduced the lengths of the skulls as measured with distances A and C at 0.1 PE and 0.3 PE (Figure 4). The width of the base of the skulls (distance B) was consistently decreased throughout all concentrations. Distance D, which is the width further toward the front of the skull, was reduced at 0.003 PE and higher. These measurement changes caused the A/B ratio to be consistently reduced throughout all concentrations. On the other hand, the C/B ratio was increased, but only at concentrations lower than 0.1 PE. The Marlboro and American Spirit exposures did not show any consistently significant patterns of cartilage-altering effects according to the measurements taken.

However, the harm-reduction Camel Blue showed significant differences in measurements A and C at 0.1 PE and 0.3 PE. Measurement D only showed a significant difference at 0.1 PE. Similar to its conventional Camel counterpart, measurement B showed a significant difference at all concentrations. These changes in measurements led to a significant difference in both the A/B and C/B ratios between 0.001 PE and 0.03 PE. Marlboro Gold, on the other hand showed, a decrease in distance B as well as distance D at the highest concentrations. These differences led to a significant decrease in the A/B ratio between 0.001 PE and 0.1 PE. The skulls of the larvae appeared to have shrunk due to the Marlboro Gold treatment, which decreased any effect on the ratios compared to Camel-treated larvae.

### 2.4. Different Tobacco Products Affect Distinct Bone Formation during Zebrafish Development

**Figure 5:**
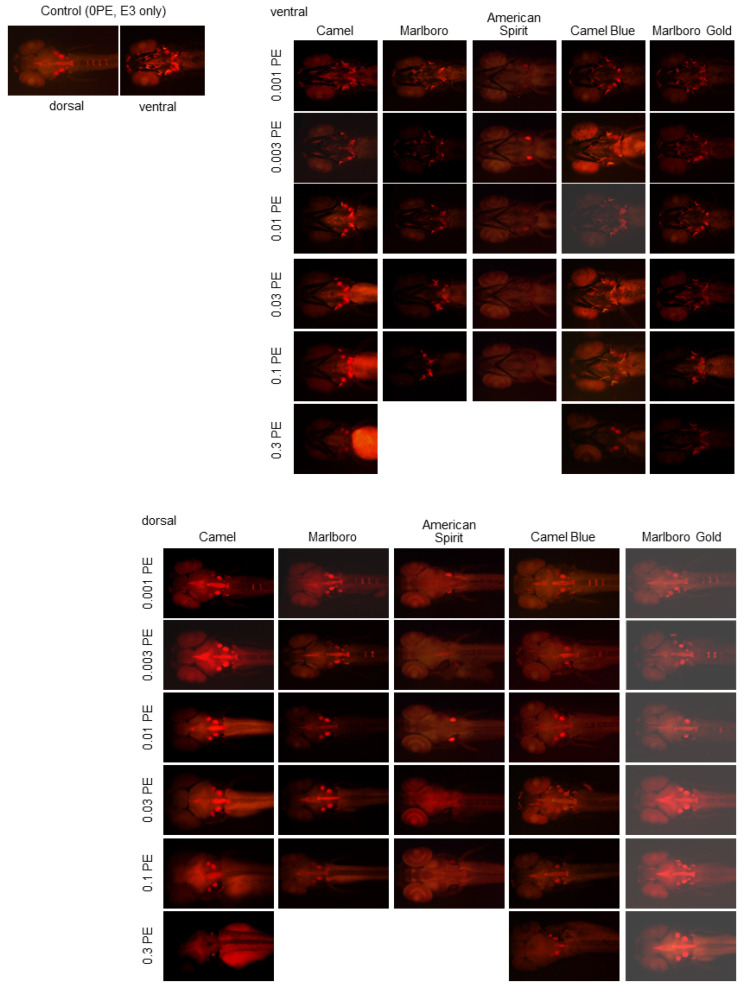
Exposure to tobacco smoke extract reduced bone mineralization in zebrafish. Embryos were placed in a tobacco-smoke-extract-containing E3 embryo media at 5 hpf, then collected, fixed, and stained with Alizarin Red S at 6 dpf. Representative images of Alizarin Red S staining assessed with a fluorescence microscope and a 532 nm filter from zebrafish larvae treated with Camel, Marlboro, American Spirit, Camel Blue, and Marlboro Gold sidestream smoke at the indicated concentrations in puff equivalents (PE) are shown.

**Figure 6:**
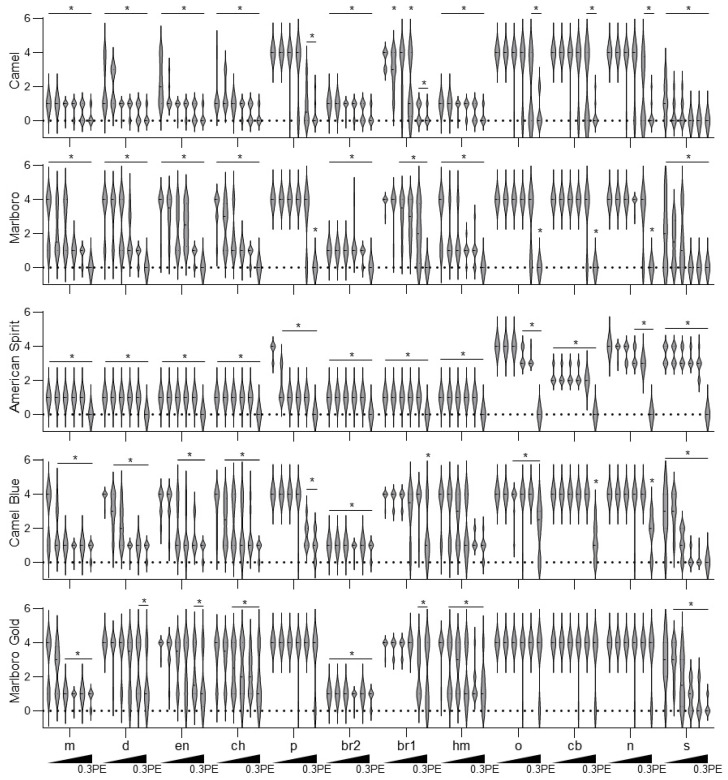
Quantitative morphometric scoring of bone mineralization defects in zebrafish larvae exposed to tobacco smoke extract. Embryos were dosed with tobacco smoke extracts at the indicated concentrations, starting at 5 hpf. Quantitative scoring was performed from images of 6 dpf-old Alizarin-Red-S-stained larvae. Bones that developed to the natural size and possessed a similar staining intensity to that of the control were given a score of 4. One point was subtracted for misshapen or dim bones, resulting in a minimum score of 2 points for a dim and deformed yet still present bone. Any bone that did not appear was given a score of 1. * *p* < 0.05, one-way ANOVA with a Dunnett’s post hoc test to correct for multiple comparisons. Br1, branchiostegal ray 1; p, parasphenoid; br2, branchiostegal ray 2; hm, hyomandibular; o, opercle; cb, ceratobranchial 5; n, notochord; s, spinal calcification.

Next, we determined whether the treatments had effects on a specific subset of bones and their degree of calcification. Skeletons were stained with Alizarin Red S and photographed (Figure 5), and then individual subsets of bones were scored according to a blind scoring system. Using this grading scale, the significance of the overall damage imposed by each of these treatments was assessed. This breakdown allowed the determination of the point at which each treatment elicited damage to skeletal tissue (Figure 6). Both Camel and Camel Blue had similar effects on targeted bones and the type of deleterious effects. At 0.1 PE and lower, the two treatments appeared to damage the same set of bones (m, d, ch, br1, p, hm, s), albeit with a higher concentration required for Camel Blue to inflict equivalent damage.

Overall, the smoke extracts from the Camel brand appeared to be more damaging to bone development than those from the Marlboro brand. Even with treatments as low as 0.001 PE, many of the smaller bones were undetected in the Camel treatment. Moreover, spinal calcification was hindered and reached a point of complete impedance at 0.01 PE and higher. The rest of the Camel- and Camel-Blue-affected bones fell into one of three categories: 1. Unaffected or nearly unaffected until 0.1 PE (p); 2. Immediate effect at 0.001 PE (d, en, ch, hm, and m); and 3. Only affected at the highest concentrations (cb, n, and o).

Similar to the Camel brand, Marlboro required a lower dosage than its “Light” equivalent to achieve similar bone developmental impedance. Both Marlboro and Marlboro Gold treatments affected the following bones: m, d, en, ch, hm, and s. Like the Camel treatment, spinal calcification was hindered and reached a point of complete obstruction at 0.01 PE and higher. Both Marlboro and Marlboro Gold showed no statistically significant decrease in bone development at the lowest PE concentration, with the exception of lowered calcification of the spinal notches with the Marlboro treatment. 

However, American Spirit was the only brand to not completely prevent spinal calcification even at the highest concentration tested. This was in contrast to every other treatment, which impeded the calcification potential of the spine at 6 dpf. The rest of the affected bones fell into one of three categories: 1. Affected at 0.003 PE or higher (m). 2. Affected mid-way at 0.01/0.03 PE and higher (d, en, ch, and hm). 3. Not affected (br1, p, o, and cb). While extracts from all products targeted most bones with equal sensitivity, some individual effects were detected for the Marlboro brand: discrete effects included the absence of p targeting in the Marlboro and Marlboro Gold treatments, a bone that was targeted by all other treatments. In turn, American Spirit was more detrimental to a wider array of bones, even at lower concentrations. In sum, our data showed that all smoke products had deleterious effects on developmental events central to bone and or cartilage formation. These facts may allow for the conclusion that there seems to be a commonality in the pathways that cause a disruption in bone formation due to exposure to tobacco products; however, there are a few nuances between the products as some treatments preserved specific bones and exasperated damage in others as outlined above.

### 2.5. Melanocyte Migration

**Figure 7:**
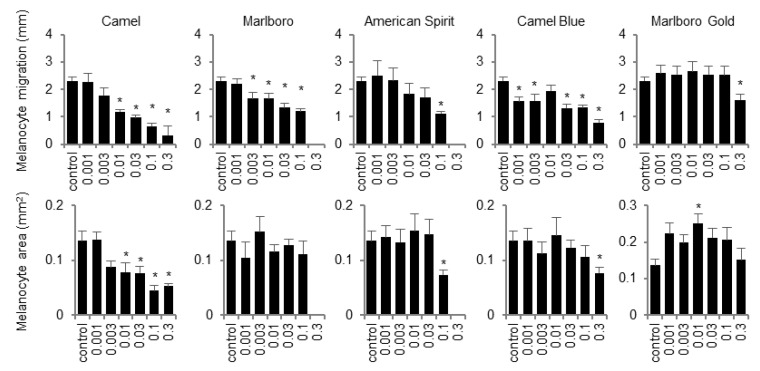
Exposure to tobacco smoke extract diminished melanocyte migration. E3 embryo media was dosed with tobacco smoke extract in concentrations as indicated at 5 hpf. The distance travelled and area of the location of melanocytes was assessed for surviving larvae at 6 dpf. X-axes represent SS smoke concentrations in puff equivalents (PE). * *p* < 0.05, one-way ANOVA with a Dunnett’s post hoc test to correct for multiple comparisons.

The identity of the affected bones scored upon exposure to tobacco products suggested that the development of the neural crest, one of the three potential lineages that can produce osteoblast progenitors, was impacted. Indeed, cigarette smoke has previously been shown to cause mutations and defects linked to inhibition of neural crest migration [27,28,29]. Neural crest migration has often been studied by measuring the distances that the neural-crest-derived pigments cells, the melanocytes, travel during embryogenesis [30,31,32]. In addition, melanocytes have been used in developmental studies for analysis of cell specification, migration, and survival [33]. In our zebrafish, the Alcian Blue staining visualized melanocytes, so we exploited this to measure their travel length and occupied area (Figure 7).

Camel exposure elicited a decrease in the travel distance and area of melanocytes between 0.01 PE and 0.3 PE. Marlboro exposure triggered a significant difference in the length of travel in melanocytes between 0.003 and 0.1 PE. Camel Blue did not cause a significant decrease in the melanocyte area, but most concentrations showed significantly reduced melanocyte travel length. In sum, the results from this study suggested that all SS smoke treatments from conventional and harm-reduction products proved detrimental to zebrafish embryo survival, early cartilage formation, and bone calcification and altered melanocyte migration.

## 3. Discussion

This study set out to determine the developmental toxicity of cigarette smoke extract exposure specifically pertaining to skeletal development in vivo. The results suggested that SS smoke inhibited bone and cartilage formation during the early stages of zebrafish development in vivo independently of the brand or harm-reduction association. While the extent of the damage to the bones and cartilage varied between the smoke from the different cigarettes, overall, harm-reduction tobacco products did not eliminate the risk associated with secondhand smoke exposure during early bone development.

Developmental toxicity may be elicited by toxicants due to the cytotoxic nature of the exposure, or because the toxicant alters cell fate trajectories in the absence of cytotoxicity [34]. Unsurprisingly, therefore, and speaking to the first possibility, all tested tobacco products caused a decline in survival rates that was more severe upon exposure to the three conventional products. The increase in exposure-mediated embryo death was accompanied by an incline in hatching difficulty. Therefore, it is indisputable that general cytotoxicity seems a contributing factor to the embryo defects observed, at least in the conventional tobacco products.

One of the immediately noticeable defects to embryogenesis triggered by exposure to SS smoke extracts were the yolk sac edemas. Since it is critical for the embryonic success and long-term health of the organism that yolk content and utilization is not compromised, the observed yolk sac edema associated with cigarette smoke extract exposure may causally link to the observed bone phenotype, although direct evidence in support of this hypothesis is lacking. Nonetheless, yolk sac edema is a common effect noted in response to toxicants of many different chemical classes [35] and is often co-observed with the spine, tail curvature, and bending [36,37,38] that was also noted here.

In addition to impacting the amount and content of deposited yolk, toxicant exposure may also affect nutrient utilization. Indeed, yolk sac edema is a commonly observed pathology in zebrafish developmental toxicity screens. A wide array of toxicants including polycyclic aromatic hydrocarbons [39,40], which are constituents found in tobacco smoke extracts [41,42], cause yolk sac edema in zebrafish embryos. While these phenotypes are not commonly observed in humans, clinical data has shown an increased rate of yolk sac edema in pregnancies that result in spontaneous abortion [43].

Our data further confirm that cigarette smoke negatively impacted neural crest development and migration, as well as mesoderm formation. Across all treatments, the majority of the bones targeted were neural-crest-derived. Furthermore, apart from the spinal segment calcification, Marlboro, Marlboro Gold, and Camel Blue did not affect any other mesoderm-derived bones, although it needs to be mentioned that most mesoderm-derived bone begin to mineralize later in development than 6dpf “Harm reduction” products either targeted the same bones as the conventional counterpart, worsened cartilage structure, or were more devastating to the zebrafish overall as observed in the American Spirit treatment. Specifically, American Spirit was unique in its preservation of the spinal calcification and showed no heart or spinal damage, but exerted the most severe damage to the neural-crest-derived bones. This notion is consistent with the smoke solutions negatively impacting the travel distance of neural-crest-derived melanocytes, which we found for all tobacco products except for American Spirit, despite the latter still affecting neural-crest derived bones.

The data presented here are in line with our previous in vitro data, which showed that SS smoke from harm reduction products, although less cytotoxic, was equally detrimental to differentiating bone cells as that from conventional products [19]. In both our previous and current study, our results were consistent with other studies that found harm-reduction products to be as harmful or more harmful to murine ESC cultures and the viability of blastomeres during the preimplantation stage of development [44,45]. Additionally, others have shown that light cigarettes are nevertheless able to induce diseases that the conventional cigarettes are known to cause, such as emphysema [46].

Research of the past two decades has shown that advertising schemes associated with the sale of “light” cigarette varieties have been very effective in making smokers believe that they are inhaling less nicotine, tar, and other harmful chemicals and therefore are significantly safer [47,48,49]. However, the literature and the internal data of tobacco companies show that smokers of “light” cigarettes crave more nicotine and will compensate by smoking more, inhaling longer and more deeply, and taking more frequent puffs [50]. Consequently, smokers that choose the “light” version of cigarettes inhale significantly more nicotine, tar, and other harmful chemicals than the amounts reported from smoke-machine data. Our study adds to this existing data set in that it suggests that even without compensatory smoking behavior, harm-reduction cigarette exposure may adversely affect skeletogenesis. This negative effect is likely still attributable to the composition of the smoke. Indeed, others have shown similar amounts of toxic heavy metal content between conventional and light cigarettes [51,52]. Indeed, cadmium, lead, and nickel alone or in combination can inhibit the calcification of mesenchymal and osteogenic cells in vitro and in vivo [53,54,55,56,57,58].

Consequently, an additional challenge in the research ahead will be the identification of the chemical in tobacco smoke that executes the deleterious outcome. Importantly, it is estimated that cigarettes contain more than 7000 individual chemicals [50]. This number of chemicals, and therefore chemical interactions, makes studying the individual effects of cigarette smoke components a difficult task. While many researchers have chosen to focus on a single component, such as nicotine, to study the resulting harm effects of exposure in cell culture or in an in vivo model, which simplifies the study of specific mechanisms for the detrimental effects seen by cigarette smoke, the compounds studied as individuals do not work in isolation and can have thousands of interactions with the other chemical compounds present in the cigarette. In fact, our own data suggested that nicotine was not the culprit chemical that induced the noted decrease in human ESC in vitro osteogenesis [19]. In other words, the additional chemical compounds may enhance or repress the function of the specifically studied tobacco constituent, highlighting the need for studies that evaluate cigarette smoke extracts as complete mixtures of all chemicals contained therein. Of note in this context is the limitation of our study to assess aqueous smoke extracts only. At the same time, however, the detection of such noteworthy skeletal malformations despite the omission of solvent-dilutable organic compounds, which are, in themselves, highly toxic, speaks for the problematic association of tobacco smoke exposure with skeletal defects.

While the adverse outcome of tobacco exposure on developing bones is clear, the molecular mechanism whereby this occurs remains unknown. Specific pathways in bone formation and bone integrity shown to be dysregulated by cigarette smoke are also essential for neural crest and mesoderm development and may become relevant in the context of the dosing scheme selected herein. For example, estrogen signaling induces neural crest differentiation and impacts the mesoderm differentiation rate [59,60,61]. Tobacco smoke also reduces estradiol [62], a hormonal activator of CTNNB1 [63,64], a transcription factor essential in proper neural crest and mesoderm development [65,66,67]. Lastly, the cellular oxidative stress elicited by tobacco exposure [68,69] may play its part in determining the deleterious outcome on the skeleton, with all cell types along the osteogenic lineage, including neural crest and mesodermal cells, being sensitive [70,71].

## 4. Materials and Methods

### 4.1. Production of Smoke Solutions

Marlboro Red 100 (named Marlboro throughout the text), Marlboro Gold, Camel, Camel Blue, and American Spirit cigarettes were obtained from a local retailer. The cigarettes were utilized to capture SS smoke puff equivalents (PE) in solution using a method previously described [19,72,73]. In brief, a smoking machine from the University of Kentucky was used to collect the smoke that burned from the tip of the cigarette for 30 min and to saturate 10 mL of E3 embryo media (5 mM NaCl, 0.17 mM KCl, 0.33 mM CaCl_2_, and 0.33 mM MgSO_4_ at pH 7). This generated SS smoke solutions at a 3 PE concentration (one PE of SS smoke was defined as the smoke produced from one minute of burning dissolved in 1 mL of medium). All E3 media components were purchased from Sigma-Aldrich, St. Louis, MO, USA. The stock solutions were filtered using a 0.2 µm Acrodisc^®^ PSF Syringe Filter (Pall Corporation, Port Washington, NY, USA) and aliquoted into sterile microfuge tubes for storage at −80 °C. Working PE solutions for exposure were made using serial dilutions.

### 4.2. Zebrafish Rearing and Collection

Adult zebrafish (strain 5D) were maintained and bred on a recirculating system with UV sterilization and mechanical/biological filtration units (Aquaneering, San Diego, CA, USA). Adult fish were kept at 28 °C and a pH of approximately 7.2 with a 14 h light and 10 hr dark cycle. Embryos were collected within 30 min of spawning, sorted, and synchronized before treatment according to previously described methods [74,75]. At 5 h post fertilization (hpf) (~30–50% epiboly), individual embryos were transferred to a 96-well plate (1 embryo per well; 8 embryos per plate; three replicate plates per exposure type) and either kept in unaltered E3 embryo media (5 mM NaCl, 0.17 mM KCl, 0.33 mM CaCl_2_, and 0.33 mM MgSO_4_ at pH 7) or in E3-containing SS smoke solution at the indicated concentrations. The plate was then covered with a lid and wrapped in parafilm. For all experiments, embryos were incubated under static conditions at 28 °C under a 14 h:10 h light:dark cycle for the entire duration of exposure.

### 4.3. Hatching Success and Survival

At 3 dpf, embryos were anesthetized at 4 °C during a ~20 min incubation and imaged using a Leica MZ10 F stereomicroscope equipped with a DMC2900 camera (Leica Microsystems Inc., Deerfield, IL, USA). After image acquisition, each embryo was recorded as having hatched from the chorion or not, relative to time-matched controls (E3 only). Based on these criteria, percent unsuccessful hatching for each treatment group was calculated.

At 6 dpf, thermal anesthesia and imaging was repeated as above. Coagulated embryos or developed embryos lacking a heartbeat were considered dead. Based on these criteria, percent survival was calculated for each group. Similarly, larvae with yolk sac edema and cardiac edema were counted and percentages of affected larvae established. After imaging, embryos were euthanized via overdose of tricaine methane sulfonate (MS-222) (Western Chemical, Inc., Ferndale, WA, USA) at 300 mg/L in embryo media.

### 4.4. Skeletal Staining

At 6 dpf, anesthetized larvae were fixed with 2% paraformaldehyde (PFA) for 1 hr at room temperature and then washed 2–3 times with storage solution (50% glycerol, 0.1% KOH). Fixed larvae were stored for up to 2 weeks in storage solution at 4 °C until further processing. Fixed larvae were then stained first with Alcian Blue (Sigma-Aldrich, St. Louis, MO, USA) solution (0.2% Alcian Blue, 0.1 M Tris-HCl pH = 7.5, and 80 µM MgCl, in 70% ethanol) overnight at 4 °C. The solution was removed and the larvae rinsed with storage solution. Larvae were bleached (1.5% H_2_O_2_, 1% KOH) for 12 min. The bleach was removed and the larvae washed 3 times for 5 min using storage solution. Larvae were then stained with Alizarin Red S (Sigma-Aldrich, St. Louis, MO, USA) solution (0.01% Alizarin Red S, 0.1 M Tris-HCl pH = 7.5, 24% glycerol) for 30–45 min. After removing the stain, larvae were washed for 5 min with storage solution and then stored in storage solution at 4 °C overnight until imaged. Additional storage solution was added as necessary if the levels dropped to prevent drying of the embryos. For imaging, Alcian-Blue- and Alizarin-Red-S-stained larvae were mounted in 80% glycerol and visualized under bright and fluorescent (532 nm) light, respectively, using a Leica M165 FC stereomicroscope (Leica Microsystems Inc., Deerfield, IL, USA).

### 4.5. Image Morphometry

Quantitative morphometric data of 6 dpf larvae were obtained with the Image J software (1.48 v). Since all images were taken with a scale bar, a scale was calibrated with the set scale tool. To do this, the straight-line instrument was selected and a line drawn along the scale bar. This defined the number of pixels per mm. For all following measurements, a randomized blind system was implemented to score staining intensity or measure distances in images (with respect to a scale bar), with the person scoring not being the experimenter. Two independent scores were obtained from two recorders, who each scored the data set two times, and averaged.

#### 4.5.1. Cartilage Lengths

The length of 4 parameters was measured from images of Alcian-Blue-stained embryos (see Figure 3 for a schematic) using the Image J measurement straight-line measurement tool, calibrated to the scale bar: A: from the left hyosymplectic (h) to the top of anterior limit (an). B: from the left hyosymplectic (h) to the right hyosymplectic (h). C: from the right hyosymplectic (h) to the top of ceratohyal (ch). D: from the right articulation (ar) to the left articulation (ar). Both A and C measurements were then compared to distance B with A/B and C/B ratios, respectively.

#### 4.5.2. Scoring of Melanocyte Travel Distance and Area

For measurements of travel distance and occupied area, the Image J measurement tool was used on lateral-view embryo images. After setting the scale, the straight-line instrument was chosen, and the melanocyte travel distance was traced along the notochord from the posterior end of the swim bladder to the last visible pigment. The measure function automatically assigned the length of the line in mm. Area was defined similarly, but instead the elliptical outline tool was used to capture width and length of the pigmented area below the notochord.

#### 4.5.3. Mineralization Abundance

Images were also utilized to quantify exposure effects on the mineralization of specific subsets of bones. Skeletons were stained with Alizarin Red S and photographed, and then the following bones were scored based on previous studies [76]: branchiostegal ray 1 (br1), branchiostegal ray 2 (br2), entopterygoid (en), maxilla (m), notochord (n), opercle (o), parasphenoid (p), ceratobranchial 5 (cb), ceratohyal (ch), dentary (d), hyomandibular (hm), and spinal segmentation (s). Bones that developed to the natural size and possessed similar Alizarin Red S staining intensity to that of the control (E3 only) were given a score of 4. One point was subtracted for misshapen or dim bones, resulting in a minimum score of 2 points for a deformed yet still present bone. Any bone that did not appear was given a score of 1.

### 4.6. Statistical Analysis

Statistical analysis was performed in GraphPad Prism (9.2., GraphPad Software Inc., San Diego, CA, USA). Normality and equal variance were examined with a Shapiro–Wilk test and Bartlett’s test, respectively. Non-normal data were log transformed before proceeding to a one-way Analysis of Variance (ANOVA) to compare the mean of each treatment with the mean of the control (E3 only). A Dunnett’s test was used to correct for multiple comparisons. *p*-values less than 0.05 were deemed significant.

## Data Availability

The datasets generated and/or analyzed in the present study are available from the corresponding author upon reasonable request.
